# Online teaching and learning: Experiences of students in a nursing college during the onset of COVID-19

**DOI:** 10.4102/curationis.v45i1.2372

**Published:** 2022-11-22

**Authors:** Lebogang L. Molefe, Nkhensani F. Mabunda

**Affiliations:** 1 Department of Nursing, Gauteng College of Nursing, Johannesburg, South Africa

**Keywords:** online teaching, online learning, best practices, interventions, challenges, students, nursing college, COVID-19 pandemic

## Abstract

**Background:**

The world has entered the Fourth Industrial Revolution. Utilisation of technology is inevitable. For the past years, the coronavirus disease 2019 (COVID-19) pandemic has halted normal operations, including in the physical classroom for nursing students. Students and facilitators had to move to a remote way of teaching and learning, utilising online teaching and learning. However, students and facilitators were not ready to use online teaching and learning. This not only resulted in numerous challenges, but also became an eye-opener for best practices and intervening strategies.

**Objectives:**

To explore and describe experiences of students in a nursing college with regard to online teaching and learning during the onset of the COVID-19 pandemic.

**Method:**

A qualitative, exploratory, descriptive and contextual research design was adopted. A purposive, nonprobability sampling approach was used to select participants from second year, third year and fourth year. First-year student nurses were excluded because they did not commence with classrooms at that time.

**Results:**

Seven themes emerged, namely knowledge, confidence, training, equipment, clinical exposure, course extension and flexibility, and all themes had subthemes.

**Conclusion:**

It is evident that students had more negative experiences during online teaching and learning than positive experiences.

**Contribution:**

The study contributed enormously to teaching and learning of student nurses in nursing colleges as its results can be used to improve nursing colleges with regard to online teaching and learning.

## Introduction

Unpreparedness of nursing colleges to effectively utilise online teaching and learning for nursing students in developing and under-developed countries remains a challenge. The Fourth Industrial Revolution (4IR) has enforced a paradigm shift of teaching and learning from a traditional method to the utilisation of online strategy. Being physically present in the classroom is not the option for learning lately because of the new era of online teaching. Online teaching involves content delivery via the Internet. The study by Oh and Yang (2019:47) affirms that the use of online teaching is an effective strategy to facilitate teaching and learning in higher education institutions. According to Álvarez-García et al. (2019:11), online learning is a teaching strategy that can be applied in the nursing curriculum because it guarantees the effectiveness of problem-based learning nursing education.

The onset of the coronavirus disease 2019 (COVID-19) pandemic further promoted the use of online teaching in nursing colleges. The pandemic globally interrupted the facilitation of the traditional classroom for teaching and learning, compelling both students and facilitators to work remotely in an attempt to curb the spread of the disease. Nursing colleges were compelled to adopt an online strategy to facilitate teaching and learning after the suspension of contact classes to control and minimise the COVID-19 pandemic virus. Online teaching supports and guides teaching and learning activities where the facilitator applies a digital curriculum (Philipsen et al. 2019:55). According to Tanis (2020:3), the best practices of online teaching and learning are that the principles must be well interpreted to provide guidance for designing the curriculum to be taught. Online teaching further encourages communication between students and facilitators in instances where assistance is required. Online teaching also promotes student-to-student interaction and discussions, because students are able to share ideas and deliberate on concepts whilst actively engaging with the content. In addition, students receive prompt feedback from facilitators, using the course management system to provide automated responses for their course, and hence give emphasis to time on task to promote active interaction on the content under discussion. Furthermore, expectations are clearly communicated to students (Tanis 2020:4). According to Harerimana and Mtshali (2018:10), the intervening conditions of online learning depend on the availability of information and communications technology setups and policies, the facilitator, students’ readiness and the training institutions’ abilities to respond to the challenges posed by a technical learning setting in undeveloped countries. Therefore, it is of significance to consider interaction mechanisms that will enhance educational and prosperous learning environments when designing online courses. The interaction mechanisms must be considered to offer enriching and thriving learning environments (Baltà-Salvador et al. 2021:7411).

This study is beneficial to nursing students, facilitators and managers of nursing colleges, as it provides insight into the current challenges faced by nursing students and facilitators during online teaching and learning. Results of the study can be used to solve challenges encountered by nursing colleges during teaching and learning, utilising online strategies. Furthermore, the study provides information pertaining to best practices and intervening conditions regarding online teaching and learning as shared by participants and, thus, enables researchers to make recommendations on how challenges can be resolved.

### Problem statement

The unpreparedness for utilising online teaching and learning during facilitation in nursing colleges is a concern that needs urgent attention. It was identified during the COVID-19 pandemic that nursing students were unable to learn because of their inability to access the Internet and a lack of digital devices to facilitate online teaching and learning. Furthermore, students and facilitators were not orientated on the use of e-learning (Crawford 2021:3; Nworie 2021:12), thus making it difficult to facilitate teaching. There was an immediate need for professional development and supply of gadgets to students and facilitators for online teaching and learning to occur; however, because of time constraints and unavailability of budget, this was not possible. It was found that nursing students were struggling to adapt to the current development of 4IR. The teaching subdivision is not yet aligned to the 4IR whereby technology is incorporated to facilitate teaching and learning. There are still challenges of resources and unconducive environments to facilitate teaching and learning online (Oke & Fernandes 2020:21). Despite challenges, online teaching and learning was found to be the only solution to facilitate teaching and learning during the COVID-19 pandemic lockdown, because it was used to support students to continue with their learning programs remotely (Hasan & Khan 2020:202).

### Aim

The study aimed to explore and describe experiences of students in a nursing college with regard to online teaching and learning during the onset of the COVID-19 pandemic.

## Method

### Research design

Researchers adopted a qualitative, exploratory, descriptive and contextual research design. This approach assists researchers to gain an understanding of the real-world context as experienced by the participants and enables researchers to obtain a detailed account of the problem of concern and further capture meaningful characteristics related to real-life events (Pelzang & Hutchinson 2018:2). Furthermore, Doyle et al. (2020:442) advised that a qualitative descriptive design is deemed most appropriate as it recognises the subjective nature of the problem, the different experiences participants have and presents the findings in a way that directly reflects or closely resembles the terminology used in the initial research question. It is against this background that the researchers adopted the design to gain an understanding of the experiences of students in a nursing college with regard to online teaching and learning during the onset of the COVID-19 pandemic.

### Setting

A public nursing college in one province of South Africa was selected for the study. It is accredited with the Council for Higher Education (CHE) and the South African Nursing Council (SANC). During a quality survey review, students and facilitators communicated their frustrations about online teaching and learning that occurred during lockdown. A few best practices pertaining to online teaching and learning were shared. Because of difficulty in accessing teaching and learning during lockdown, students were found to be incompetent to be promoted to the next year of study after lockdown. Measures to prevent COVID-19 infections were continued even though all restrictions were lifted. This included keeping a social distance of 2 m apart, sanitising hands regularly with 80% alcohol-based sanitiser and wearing of facial masks for those with influenza symptoms.

### Population and sampling

A purposive, nonprobability sampling approach was used to select participants. Baltes and Ralph (2021:3) explained purposive, nonprobability sampling as a technique that allows the researcher to select information-rich cases that contribute much to the central focus of the study. Nursing students were selected as a population of the study because they were the relevant people to share their experiences with regard to online teaching and learning during the onset of the COVID-19 pandemic. The population was further refined to an accessible population; hence, one nursing college was used. Participants were recruited from second-year, third-year and fourth-year nursing students. Participants were recruited verbally by visiting them in classes during study periods. The study purpose, risks and benefits were verbally explained to them, including the advice that participation is voluntary. Twenty-two nursing students agreed to participate in the study. Three groups were formed from the 22 students. The first group had seven members, inclusive of three men and four women. The second group had seven members, inclusive of two men and five women; and the third group had eight members, inclusive of two men and six women. Participants were from diverse cultural backgrounds; however, all understood English and agreed to be interviewed in English. All participants were 18 years and above and signed voluntary consent forms to participate. Participants were second-year, third-year and fourth-year student nurses. First-year student nurses were excluded because during the onset of the COVID-19 pandemic, they had just commenced with the course and were still in the orientation period; hence, classes had not commenced at that time.

### Data collection

Data were collected through focus group interviews. Three sets of interviews were conducted, as there were three groups. The two researchers collected data, with one researcher leading focus group interviews for the two groups and another researcher interviewing the last group. The two researchers worked together and both agreed that one would interview two groups, whereas another would interview one group. However, it should be noted that the two researchers are working in another nursing college which was not part of the setting for this study. Therefore, they both did not have any relationship with participants. Each group was interviewed on a different date and time. The first group was interviewed on 14 June 2022, the second group on 21 June 2022 and the last group on 29 June 2022. Between March 2020 and February 2022, the college was utilising online teaching because of hard lockdown. In March 2022, the college resumed face-to-face classroom teaching and learning. One researcher interviewed two focus groups and another researcher interviewed one focus group. A classroom was booked for the interviews. All participants were seated comfortably in chairs. The classroom was neither cold nor hot, and there was enough ventilation and lighting. On the day of the interview, a sign reading ‘do not disturb, interview in process’ was pasted outside the door. One interview lasted for 40 min, the second interview took 34 min and the third interview took 28 min. No interview exceeded 1 h. The following process was followed: a welcome statement was given by the researchers, followed by an overview of the topic. The researchers outlined ground rules during the process:

- One person must talk at a time.
- One should not interrupt another member when talking.
- No judging the views of another person, as there is no right or wrong information.
- Please take turns in talking.
- Do not all speak at once.
- There is no need to agree with others.
- One must listen respectfully when others share their views.
- Cellular phones must be turned off.
- Participants’ responses will be audio-taped as agreed.

The researchers posed the following grand-tour question to participants: what are your experiences with regard to online teaching and learning during the COVID-19 pandemic? The researchers minimally interrupted participants, allowing them to share their experiences freely. The researchers further used the following probing questions to get clarity: ‘would you explain further’? ‘What do you mean?’ ‘I do not understand’. An audio tape recorder was used to record participants’ verbal responses. All participants gave verbal and written consent for the usage of an audio tape recorder. Field notes were captured and recorded. All participants were very much free during discussions and did not show any sign of discomfort. Both researchers agreed and ensured that they collected data until data saturation, that is, until no new information emerged from all members of the group. Researchers managed to ascertain data saturation because participants started to repeat the same information, indicating that there was no new information.

### Data analysis

Researchers utilised content analysis as a method to analyse data. Content data analysis is a process that assists to extract the essential meaning of the topic from the verbal and written versions of the recorded data, separate them into constituent concepts and arrange them into themes, subthemes and categories (Polit & Beck 2021:564). Each researcher analysed the data collected by them, following Creswell’s six steps of data analysis (Creswell 2013). Researchers listened to the tape and transcribed data. Researchers further reviewed field notes. Transcripts were read and reread, and reports of the individual focus groups were prepared in a question-by-question format with amplifying quotes. Codes were written next to the appropriate segments of the text. The most descriptive wordings for the topic were found and turned into categories. Open coding was conducted paragraph by paragraph. Codes were reviewed, revised and combined into themes and subthemes. Data were organised in such a way that themes and subthemes were presented in a cohesive manner. Researchers further utilised an independent co-coder who also analysed data, following the same format. The purpose of choosing an independent coder was to avoid bias by researchers and also to ensure that results are not shaped by the researchers’ own views. Following independent data analysis by the researchers and co-coder, a virtual meeting was convened amongst the three to discuss findings. Where there were disagreements on themes and subthemes, a discussion was made and consensus on the final themes and subthemes was reached.

### Trustworthiness

Korstjensa and Moserb (2018:121) explained trustworthiness as the quality of being extremely thorough and careful, and it simply poses the question, ‘can the findings be trusted?’ According to Korstjensa and Moserb (2018:121), five testing criteria for trustworthiness in a qualitative study need to be observed, namely credibility, dependability, confirmability, transferability and authenticity. Credibility was attained through prolonged engagement with participants and member checking. Furthermore, persistent observation, triangulation, negative case analysis, referential adequacy and investigative procedures were carried out. Dependability was attained through audit trail and the use of a co-coder during data analysis. The audit trail established and conducted by researchers was monitored for verification by the supervisor of the corresponding author, who is a doctoral student at North-West University. Researchers were transparent from the start of the research to the reporting of findings. Confirmability was attained by ensuring that the findings were not shaped by bias, motivation or interest; hence, they ensured bracketing and engaged in triangulation. Researchers further used a co-coder. Transferability was attained through thick description of the research methodology, which gives other readers a sense of the complexity of the reality. Authenticity was attained by ensuring that participants were fairly selected through conforming to exclusive and inclusive criteria, as explained under the heading ‘population and sampling’. The researcher ensured that the report conveyed participants’ feelings and not their own feelings.

### Ethical considerations

This article forms part of the doctoral degree study for the corresponding author, who is writing a thesis in article format. The corresponding author therefore has a valid ethical clearance certificate obtained from the North-West University Health Research Ethics Committee (NWU-HREC; reference number NWU-00462-20-A1). The certificate allows the researcher to conduct a qualitative study on human beings. Permission to conduct the study was further obtained from the chairperson of the ethics committee of the campus, in agreement with the principal of the nursing campus, after researchers explained *Curationis*’s call for submission of papers specifically for selected topics as outlined. Researchers identified the relevant topic applicable to the campus: ‘Online teaching and learning: Experiences of students in a nursing college during the onset of COVID-19’. Researchers performed critical assessment of the status of online teaching and learning of the college and found the need to conduct the study in order to understand experiences of students in a nursing college with regard to online teaching and learning during the COVID-19 pandemic. A permission letter signed by the chairperson of the ethics committee is available, dated 10 June 2022. Participants gave voluntary consent to participate and agreed to be audio-taped. Participants’ names were not used anywhere, but codes were used (e.g. P-A). All participants were interviewed after all COVID-19 restrictions were lifted.

## Results

Three groups of nursing students were interviewed, with the first group having seven members, the second group having seven members and the third group having eight members. The total number of students who participated was 22. Participants were of different age categories as outlined in Table 1.

**TABLE 1:** Demographic information of participants.

Number	Participant number	Age (in years)	Gender	Title
**Group one**				
1	P-A	19	Female	Student
2	P-B	23	Male	Student
3	P-C	25	Female	Student
4	P-D	20	Male	Student
5	P-E	24	Female	Student
6	P-F	20	Female	Student
7	P-G	18	Female	Student
**Group two**				
1	P-H	20	Female	Student
2	P-I	22	Female	Student
3	P-J	20	Female	Student
4	P-K	19	Male	Student
5	P-L	28	Female	Student
6	P-M	26	Female	Student
7	P-N	18	Male	Student
**Group three**				
1	P-O	23	Female	Student
2	P-P	18	Male	Student
3	P-Q	24	Female	Student
4	P-R	19	Female	Student
5	P-S	29	Male	Student
6	P-T	26	Female	Student
7	P-U	19	Female	Student
8	P-V	22	Female	Student

Seven themes and their subthemes emerged and are given in Table 2 and further explained.

**TABLE 2:** Themes and subthemes.

Themes	Subthemes
Knowledge	Lack of knowledge Lack of orientation
Confidence	Increased confidence
Training	Lack of training
Equipment	Unavailable gadgets Lack of provision of data
Clinical exposure	Lack of clinical exposure
Course extension	Inability to submit assessments Inability to meet required clinical hours
Flexibility	Environmentally friendly

### Theme 1: Knowledge

Knowledge sharpens our skills and helps the brain to function smoothly and effectively, hence students most often associate the need for knowledge with spontaneous learning situations (Skarstein & Skarstein 2020:320).

#### Subtheme 1.1: Lack of knowledge

A lack of knowledge promotes brain dysfunction, leads to poor comprehension of information and ultimately results in a lack of learning (Skarstein & Skarstein 2020:321). The statement is affirmed by participants of the study, who cited that lack of knowledge regarding online learning resulted during the COVID-19 pandemic. The following quotes were cited by participants:

‘I never heard anything about online learning until we were on lockdown.’ (P-C, female, 25 years old, student)

‘I received WhatsApp communication, instructing me to log in tomorrow at 09h00 because classes are resuming online. I was stressed because I did not know anything about online learning.’ (P-F, female, 20 years old, student)

‘This was so shocking and disturbing that I had to start something I did not know’. (P-T, female, 26 years old, student)

‘Yes, I was told to log in and attend classes, otherwise I will be zero-rated if I do not log in and I will have to repeat the level’. (P-N, male, 18 years old, student; P-V, female, 22 years old, student; P-H, female, 20 years old, student; P-O, female, 23 years old, student; P-R, female, 19 years old, student)

Lack of knowledge became a stressor to participants and frustrated them greatly. Stress and frustrations from participants are attested by the following quotes:

‘I did not even know how to log in, where to start, it was just so frustrating and very stressful.’ (P-Q, female, 24 years old, student)

‘I had to press my phone anywhere, in case I become luck and press the button that will enables me to log in and attend class. I ended up crying because I could not log in, therefore missing the information.’ (P-F, female, 20 years old, student)

‘My mother found me crying and I could not even tell her the reason I am crying because I did not want to frustrate her as well’. This college is failing us, how can they expect us to be competent with online learning when we were never taught about it prior COVID-19? It is like they want to see us failing and repeating the class. (P-B, male, 23 years old, student)

The sentiment and frustration was similar for most participants:

All of the responses affirmed that indeed students did not have knowledge regarding online learning.

#### Subtheme 1.2: Lack of orientation

Deficiency in orientation may result in a lack of clarity about the tasks and responsibilities, causing role ambiguity, which is a stressor. When a person experiences role ambiguity, he or she is prevented from achieving his or her personal goals because of a lack of direction, information, resources or any combination thereof (Raub et al. 2021:2). The statement was attested by participants of the study, who indicated that they were not orientated on online learning. The following quotes attest this:

‘I was not orientated on online teaching. None of my colleagues were orientated. The college needs to orientate us on online teaching and learning before we can be expected to use it because some of us did not do IT at all, therefore it becomes difficult for us.’ (P-V, female, 22 years old, student)

The lack of orientation was further affirmed by another participant, who said:

‘They just throw us in in a deep end without at least putting us through on the use of online learning and I feel that it is very much unfair. Perhaps they need to introduce a fundamental module that will teach us something about e-learning.’ (P-V, female, 22 years old, student)

Another participant said:

‘IT [*information technology*] people employed to assist lecturers with connections during classroom sessions must be given a one week a period so that they orientate us and lecturers on how to use our cellphones and computers for online learning.’ (P-M, female, 26 years old, student)

One participant further said:

‘I was at the university but I only did first year. I had to drop-out because of funds. There at university, there is computer laboratory and students have periods allocated to go to the laboratory and practice. Why can’t the college do the same thing?’ (P-E, female, 24 years old, student)

Based on the participants’ responses, it is clear that there was no orientation for students about online learning, and this contributed to students’ frustrations. Furthermore, there was no computer laboratory where students can be introduced to e-learning.

### Theme 2: Confidence

Confidence is a feeling or belief that one can have faith in or rely on something, and it comes from feelings of well-being, acceptance of your body and mind and belief in your own ability, skills and experience (Wendy 2017:108). The subtheme that emerged for confidence is increased confidence, which is explained.

#### Subtheme 2.1: Increased confidence

The subtheme is explained in alignment with explanation of confidence in the theme. Although most participants of this study cited challenges to online teaching and learning, some participants indicated that they felt confident after utilising online learning, as attested by the following quotes:

‘Much as I did not know anything about online learning initially, I started to enjoy if after some days because I am personally a shy person in class. However, during online teaching and learning, I find myself participating in debates and discussions without fear and that boosted my self-confidence.’ (P-G, female, 18 years old, student)

‘I started to enjoy on the second week because I had to learn very fast. I also liked the fact that I did not have to wake up in the morning and travel to campus. It saved me travelling money, although I did spend a bit on buying data.’ (P-A, female, 19 years old, student)

With reference to this, it is evidenced that online learning has positive results if well applied.

### Theme 3: Training

Training represents a good opportunity for employees to grow their knowledge base and improve their job skills to become more effective. It further influences employee performance and makes possible the development of new skills and knowledge (Sendawula et al. 2018:9). The subtheme for the theme training is the lack of training, which is explained.

#### Subtheme 3.1: Lack of training

The subtheme of the lack of training is explained in line with the information on training. Participants cited that during online teaching, they realised that facilitators themselves were not trained on online teaching. The following quotes attest to this:

‘The lecturer herself was not sure how to bring students together for discussions during online learning. She was really struggling to control students. She could not see us when we have raised hands to ask questions or to answer questions. I think lecturers themselves need further training.’ (P-F, female, 20 years old, student)

‘One day we started class very late because the lecturer could not manage to log in herself and had to call an IT to guide her on how to log in.’ (P-N, male, 18 years old, student)

‘I was not comfortable that an IT personnel had to be part of our class, I was hoping that she will only assist the lecturer to connect, then exit the class. However, she had to be part of us throughout and continue to guide the lecturer on what to do because the lecturer did not know what to do. Lecturers need more training themselves.’ (P-P, male, 18 years old, student)

Responses as cited by participants clearly prove that facilitators themselves were not trained on online teaching and learning. Participants further recommended regular in-service trainings for lecturers.

### Theme 4: Equipment

Lockdown and social distancing measures because of the COVID-19 pandemic have led to closure of training institutions and higher education facilities, thus compelling a paradigm shift to e-learning. In conducting e-learning, institutions had to ensure that e-learning tools are available during the closure of such institutions, because without such tools, both facilitators and students struggled in online teaching and learning (Pokhrel & Chhetri 2021:135). Two subthemes of this study, unavailable gadgets and lack of provision of data, addressed the issue of equipment at the college where the study was conducted, and they are explained.

#### Subtheme 4.1: Unavailable gadgets

Gadgets in the learning environment play an important role as resources of learning and support the process of learning. Furthermore, research has shown that the use of gadgets in class can aid learning, especially for students with special needs or those having difficulty in learning. Lack of such can be a stressor, especially in the changing world where technology is growing faster (Ratnasadi 2019:25). The statement is affirmed by participants of the study and attested by following quotes:

‘We were not given laptops or data to log in. One had to make a plan to log in, such as purchasing data from own pocket, borrowing laptops from friends or family members, or requesting money from parents to buy data.’ (P-B, male, 23 years old, student; P-F, female, 20 years old, student)

The lack of gadgets further frustrated students during the COVID-19 lockdown:

‘It was worse because for some of us, our parents are pensioners and there is nobody else at home who is working, therefore, parents had to give us pension money to buy data because they were afraid that we will fail.’ (P-I, female, 22 years old, student)

‘We were frustrated during lockdown. It was not funny at all. We did not have laptops or data to log in. Our cellphones are not smart phones.’ (P-M, female, 26 years old, student; P-O, female, 23 years old, student; P-Q, female, 24 years old, student)

Participants further mentioned that those who did not have smart cell phones were completely lost because they missed out on all content that was delivered during lockdown:

‘I did not have a smart cellphone and therefore could not access online classes.’ (P-U, female, 19 years old, student)

‘I made peace with the fact that I will repeat a level next year because I lost information given during online learning.’ (P-T, female, 26 years old, student)

Financial challenges did not allow participants who did not have smart cell phones (or even laptops) to purchase them:

‘My parents are unemployed and could not even assist me to purchase a smartphone or a laptop so that I can learn. I could see that they are more affected than myself and I was worried that they will end up with depressions and strokes.’ (P-L, female, 28 years old, student)

It is therefore evidenced that unavailability of gadgets for learning was a big challenge because it disadvantaged students in terms of learning.

#### Subtheme 4.2: Lack of provision of data

A study by Chase et al. (2018) highlights that the challenges regarding learning can be addressed through the availability of technological gadgets and Internet connectivity. Furthermore, such gadgets and Internet connectivity have shown a positive effect on students’ perceived efficiency of working (Chase et al. 2018:5). It is therefore evident that a student who does not have data for Internet connectivity will struggle. This will even be worse for a facilitator who is expected to offer online teaching without data. It confirms that teaching and learning can be a failure. The statement is attested by quotes from participants, who said:

‘It was not only us students who did not have data. Lecturers also indicated that they are not provided with data, therefore are forced to use their own data.’ (P-A, female, 19 years old, student; P-C, female, 25 years old, student; P-F, female, 20 years old, student, P-H, female, 20 years old, student)

Participants further mentioned that lecturers had to resort to other means to communicate to students, such as sending WhatsApp messages to students. This is attested by the following quotes:

‘Other lecturers would send a WhatsApp message prior class to say that they have insufficient data to log in, therefore, class for the day will be facilitated via WhatsApp.’ (P-J, female, 20 years old, student; P-N, male, 20 years old, student; P-O, female, 23 years old, student, P-P, male, 18 years old, student; P-R, female, 19 years old, student; P-T, female, 26 years old, student; P-U, female, 19 years old, student)

The lack of data to access Internet connectivity compromised teaching and learning and encouraged fewer discussions and interactions.

### Theme 5: Clinical exposure

It is imperative for nursing students to employ their knowledge and skills in clinical environments to acquire the required qualifications for taking care of patients, and their success depends to a great extent on efficient clinical training and exposure (Kalyani et al. 2019:2). The subtheme for this theme is lack of clinical exposure and is explained.

#### Subtheme 5.1: Lack of clinical exposure

All participants of this study cited that they did not receive clinical exposure during lockdown because they were not allowed to have physical contact with patients. This resulted in them not meeting expected clinical hours as outlined by SANC. Not meeting requirements for clinical hours enormously affected their training because participants did not feel confident to provide quality nursing care. The statement is attested by the following quotes:

‘We could not be placed in clinical facilities to apply theory to clinical. We could not engage with patients. We are not sure if we can safely provide quality care to patients. We were not even assessed for competency in clinical skills. What we did is just theory, no clinical exposure. SANC will not allow us to proceed to the next level without relevant clinical hours for this level.’ (P-S, male, 29 years old, student)

The statement is an indication that students were unable to relate theory to clinical practice.

### Theme 6: Course extension

Nursing students’ academic failure is a phenomenon of growing international interest. Factors identified as influencing nursing students’ failure, which leads to course extension, include availability of facilities such as facilities and equipment relevant to facilitate e-learning, amongst others (Dube & Mlotshwa 2018:1851). Two subthemes emerged: inability to submit assessments and inability to meet required clinical hours. The two subthemes are explained.

#### Subtheme 6.1: Inability to submit assessments

Because of various challenges cited by participants regarding online teaching and learning, it was difficult for participants to even submit assessments online. The statement is attested by the following quotes from participants:

‘I missed deadline for submission because I tried to submit but the system did not allow me. I could not understand what the problem was.’ (P-C, female, 25 years old, student; P-E, female, 24 years old, student)

Some participants cited that uploading an assignment to the system was very challenging and frustrating. One participant said:

‘I uploaded my assignment but it could not go through as it was saying my details are wrong, yet I knew that my details are correct. Iyoo ma’am, I felt like crying.’ (P-S, male, 29 years old, student)

Some participants further complained of time allocated to submit assignments online and said:

‘With online tests, you need more time. However, we were given the same time as when we are writing in class. We felt that it was very much unfair.’ (P-F, female, 20 years old, student; P-V, female, 22 years old, student)

This indeed confirms that more time needed to be allocated for enabling students to effectively submit their assessments.

#### Subtheme 6.2: Inability to meet required clinical hours

Participants cited fear of not progressing to the next level because of unmet clinical hours. One participant said:

‘We are afraid that we will be expected to repeat the level because we do not meet the minimum requirements to be promoted to the next level.’ (P-F, female, 20 years old, student)

Another participant said:

‘You know that SANC is very strict when coming to clinical hours, therefore, I do not see us moving to the next level. I am waiting for us to be called so that they inform us that our course will be extended so that we are placed in clinical areas to be able to meet the required hours.’ (P-U, female, 19 years old, student)

Inability to meet clinical hours required for progression is a reason for eminent and unavoidable course extension because of SANC minimum requirements for clinical practice. This statement is affirmed by one participant, who said:

‘I heard from the grapevine that our course will be extended and that the new master plan will be provided to us very soon. Apparently the revised master plan will allow us to be placed in clinical facilities.’ (P-S, male, 29 years old, student)

All of the participants’ citations are evidence that course extension is eminent and unavoidable. Even though online teaching and learning had some positivity in terms of a theoretical component, it did not allow students to have practical exposure to clinical care, which is a requirement by SANC; thus, they were disadvantaged on the clinical component. Without clinical exposure, students cannot proceed to the next level.

### Theme 7: Flexibility

Online teaching and learning is flexible. It helps with easy administration and accessibility, along with less use of resources and time. Students can easily access learning materials. Furthermore, students become self-directed learners, which encourages lifelong learning (Mukhtar et al. 2020:28). A subtheme, environmentally friendly, emerged and is explained.

#### Subtheme 7.1: Environmentally friendly

Online teaching and learning is environmentally friendly because there is no need to print question papers, and students do not need to write, thus avoiding printing and writing errors and even saving money as no printing of papers and buying of answer sheets are required. It saves time because it allows less time between setting of the assessment and content delivery for facilitators because students can complete much of their coursework at home and only requiring in-person attendance a few times per week, thus increasing more time for marking of assessments by facilitators. This statement is in line with the sentiment by Mukhtar et al. (2020) and is further attested by participants’ quotes:

‘Online teaching and learning technique is far much better than writing in a paper. It saves time as one does not need to write, but to type.’ (P-D, male, 20 years old, student)

‘The computer would underline the word that was incorrectly spelled for me and provided me with correctly spelled word. It was so amazing.’ (P-I, female, 22 years old, student)

‘Writing takes time, hence I don’t normally finish writing test. But this time around, I managed to finish as I was just typing and submitting. With online, you just type, click and send.’ (P-J, female, 20 years old, student)

Another participant said:

‘In comparing online assessment with traditional assessment, I prefer online assessment. I suppose if we can be orientated and trained thoroughly before, online assessment is the future.’ (P-M, female, 26 years old, student)

Participants’ quotes indicate that online teaching and learning is the best technique for student nurses; however, more training and orientation are required before implementation.

## Discussion

This study on online teaching and learning during the onset of the COVID-19 pandemic gives insight on what students experienced during hard lockdown, where it was not possible to facilitate teaching and learning through the traditional ways of the physical classroom. Furthermore, the study provides best practices and intervening conditions surrounding online teaching and learning. Seven themes emerged, namely knowledge, confidence, training, equipment, clinical exposure, course extension and flexibility. When a person is not orientated and does not have knowledge of what she or he is expected to do, the activity to be performed becomes a failure. A study by Wijayanti (2019:455) affirms that knowledge and orientation are effective in improving performance because they lead to behavioural changes as a result of understanding and experience gained during training. Furthermore, knowledge and training improve an individual’s knowledge and skill. In addition, Lee (2018:3) emphasises that knowledge is the primary source of competitive advantage and is critical to the long-term sustainability and success.

Self-confidence enables the person to overcome problems that may arise in life. People who are confident are optimistic and willing to succeed, do not resist difficulties, are comfortable and affectionate in human relations, are open to new thoughts and experiences and have progressive personality. They have a sense of self-respect, being useful, important people who are worthy of respect and acceptance. Low-confidence people find themselves unsuccessful and worthless because they believe that they cannot solve problems in life. They experience the stress and anxiety of constant desperation (Illan & Bardakci 2020:112).

Training is a key strategy to ensure a person becomes confident in what she or he does, whether at work or educational study. Inadequate training promotes poor performance and increases the level of stress. A lack of training encourages unhappiness. For a person who is studying, a lack of training leads to poor progression, thus decreasing developmental opportunities. Students who are not properly trained on what to do to enhance their study produce less work and a lower quality of work, and subsequently their progression is poor (Sendawula et al. 2018:12).

Equipment is necessary to perform any activity. Without equipment, a person cannot perform an activity. A study by Basit, Quaratulain and Hafeez (2021:355) affirmed that technological equipment such as laptops, smartphones, iPads and Internet are key for teachers and students in higher education because they provide assistance to scholars for dynamic learning during class, during lectures and outside class. They also allow the facilitator to deliver knowledge that meets the requirements of the present information technology era. Therefore, the lack of such devices discourages superiority of learning, critical thinking and communication skills. Furthermore, it ensures that learning does not occur. When schools provide students with technological devices to use in classroom and at home, learning improves. Therefore, students who do not have such devices are at risk of performing poorly, failing modules and standing a high chance of terminating the course because of frustrations. This sentiment is echoed by Day, Fenn and Ravizza (2021:e0251792).

Clinical exposure for student nurses is very important. It assists students to become competent in taking care of patients (Kalyani et al. 2019:2). There are minimum hours expected to be accomplished by students. Therefore, students are expected to be placed in clinical facilities to have direct contact with real patients. The lack of clinical exposure for a student nurse has a negative result because it does not prove that a student is competent and ready to proceed to the next level. A study by Zulu, Du Plessis and Koen (2021:a1615) affirmed that clinical placement of student nurses exposes them to learning opportunities for the acquisition of clinical skills as students are able to transfer classroom knowledge to clinical practice, because after completion, they will deal with lives of human beings. Therefore, when a student did not have clinical exposure during training, it is not guaranteed that such a student can be competent in the provision of care and saving lives.

Course extension, which can result when a student did not meet requirements to proceed to the next level, such as a lack of clinical exposure, a lack of knowledge or insufficient training hours required by the SANC and CHE, has been found to be a challenge in this study. A study by Motsaanaka, Makhene and Allay (2020:a1217) affirmed that a lack of clinical placement during the training of student nurses and the lack of acquisition of theoretical and practical hours for student nurses as stipulated by the SANC will prohibit a student from registering as a professional nurse, because such a student is seen as lacking the knowledge to provide quality care and is incompetent in clinical skills. It is therefore imperative for a student to have knowledge and skill expected. Based on this, it therefore makes sense why a student who does not meet minimum requirements to proceed to the next level can be subjected to course extension.

Flexibility is a motivation strategy that can help an individual to improve performance and boost self-confidence. A flexible learning environment helps students to achieve greater study–life balance, increases performance and students’ satisfaction and further improves morale. A flexible environment further reduces stress, improves productivity and promotes learning. Recent studies have shown that flexible teaching and learning gives students a choice in what kind of learning space works best for them and helps students to work collaboratively, communicate and engage in critical thinking. Students become happier, engage more and improve in academic performance overall. Adaptive flexibility enables a person to shift his or her perspective on an occurring problem and to use new strategies if the existing methods no longer work (Lubke, Pinquart & Schwinger 2021:4).

### Limitations

All participants were from the same college. Other colleges were not covered because of the unavailability of permission letters to conduct studies in those campuses. Therefore, it is possible that challenges, best practices and intervening conditions can be different in those colleges. Because of the qualitative nature of the study, the findings of this study cannot be generalised to other colleges; however, they can be used to improve conditions in those colleges.

### Recommendations

Recommendations address challenges to online teaching and learning, best practices and intervening conditions. Recommendations are discussed under the headings of recommendations for practice, policy development and research.

#### Recommendations for practice

The researchers recommend that online teaching and learning be utilised more even after the pandemic so that students can acclimatise themselves easily. The researchers further recommend regular in-service trainings for students and facilitators on online teaching and learning. Managers of nursing colleges and the Department of Health must ensure that students and facilitators are provided with gadgets and data for online teaching and learning on an ongoing basis. Therefore, an increase in budget by the Department of Health for nursing colleges is necessary. Online teaching and learning does not expose students to real clinical settings wherein students have the chance to practically provide care to real patients. Researchers therefore recommend implementation of innovative teaching strategies that can accommodate clinical exposure. With regards to best practices and intervening conditions, it is imperative for managers, educators, the Department of Health, community representatives, SANC, CHE and all stakeholders to meet, discuss, deliberate and reach consensus on how best they can accommodate clinical practices using online teaching and learning.

#### Recommendations for policy development

The researchers recommend a review of current policies on the education and training of student nurses so that they become aligned to the 4IR where technology is key. The researchers further recommend the development of new policies that will take cognisance of online teaching for both theoretical and clinical components.

#### Recommendations for research

The researchers recommend future research studies focusing on clinical teaching using online teaching. Furthermore, future studies should address how best the SANC clinical hours can be accomplished without a student having to be present physically with a patient and without compromising the quality standards of care.

## Conclusion

The study explored and described experiences of students in a nursing college with regard to online teaching and learning during the onset of the COVID-19 pandemic. Results revealed that during hard lockdown, students had more negative experiences with online teaching and learning than positive experiences. Supporting this are seven identified themes, namely knowledge, confidence, training, equipment, clinical exposure, course extension and flexibility. From these seven themes, only two themes address positive experiences, namely confidence and flexibility. Recommendations on how to further enhance positive experiences regarding online teaching and learning were made. Recommendations were discussed under the headings of recommendations to practice, policy development and research. Findings of this study therefore contribute enormously to teaching and learning environments in nursing colleges because they can be used to improve online teaching and learning, instil positive attitudes regarding online teaching and learning and increase students’ and lecturers’ morale.
